# Editorial: Advanced biophysical and biochemical technologies to study GPCR signal transduction

**DOI:** 10.3389/fendo.2023.1354689

**Published:** 2024-01-04

**Authors:** Maria Majellaro, Alexey Bondar

**Affiliations:** ^1^ Celtarys Research, Santiago de Compostela, Spain; ^2^ Faculty of Science, University of South Bohemia in Ceské Budejovice, Ceské Budejovice, Czechia; ^3^ Laboratory of Microscopy and Histology, Institute of Entomology, Biology Centre of the Czech Academy of Sciences, Ceské Budejovice, Czechia

**Keywords:** GPCR, G protein, cannabinoids, serotonin, microscopy

In the last few decades, our understanding of druggable and undruggable proteins involved in pathological processes has significantly expanded, thanks to advancements in computational technologies and bioengineering (1, 2). These advancements have propelled the progress of sophisticated biophysical and biochemical methodologies employed in assay development and target characterization across a diverse array of targets.

This Research Topic is focused on one of the major classes of target membrane proteins: G protein-coupled receptors (GPCRs) (3). Approximately 34% of approved drugs target GPCRs, with more than 800 different subtypes identified (4). While some GPCRs are well-characterized through various techniques such as X-ray crystallography, CryoEM, and NMR, the structures or ligands of others remain completely unknown, as is the case with orphan receptors. This circumstance underscores the pressing need to develop general methodologies for the systematic characterization of these receptors and the corresponding identification of hits (5).

This Research Topic has been conceived and conceptualized through a collaboration between Frontiers in Endocrinology and the European Research Network on Signal Transduction (ERNEST) COST Action. The primary objective of ERNEST is to create a comprehensive holistic map of GPCR signal transduction, with the ultimate goal of supporting the pioneering concept of developing chemical modulators that act on specific pathways. The articles included in this Research Topic – spanning from original research articles to review- highlight significant breakthroughs in the field, with a particular focus on serotonin, cannabinoid, dopamine, and glucagon-like peptide 1 (GLP-1) cellular signaling, all of which are mediated by GPCRs. The included publications present new methodologies based on utilization of fluorescent ligands and delve into understanding the role of specific GPCRs in pathophysiological conditions.

Advancing innovative methodologies for the identification of new hits and leads is a crucial facet in catalyzing the GPCR drug discovery process, and this constitutes the primary focus of the article authored by Tahk et al. The team conducted a thorough characterization of the D3 dopamine receptor, utilizing a novel and versatile fluorescent probe. This enabled the development of competition binding assays employing fluorescence polarization. Notably, the study was conducted within a distinctive biological matrix—baculovirus particles overexpressing the D3 dopamine receptor on their surface. Furthermore, the researchers established quantitative epifluorescence microscopy for D3 receptors in live cells.

The review by Durydivka et al. describes the role of an underappreciated factor Src homology 3-domain growth factor receptor-bound 2-like endophilin interacting protein 1 (SGIP1) in regulation of cannabinoid signaling. Quite intriguingly, SGIP1 is capable of preventing internalization of presynaptic cannabinoid receptors type 1 (CB1R) and likely plays a key role in regulation of the endocannabinoid system signal transduction. The review presents potential avenues for modulating CB1R-SIGP1 interaction and targeting the therapeutic potential of the cannabinoid system.

The original research article by Buo et al. determines the role of serotonin (5-HT) in the neuroendocrinal regulation of ovulation in the central nervous system. The publication provides an exemplary instance of utilizing calcium imaging and electrophysiology on mouse-derived brain slices. Their study showcases the involvement of the 5-HT2 receptor in the preovulatory luteinizing hormone surge through kisspeptin neuron activation.

Finally, the article by Hernández-Montoliu et al. expands the scope of the implications of GPCRs in pathological domains, correlating GLP1 and GLP2 secretion with improved metabolic control in patients with type 2 diabetes after metabolic Roux-en-Y gastric bypass (mRYGB). Quite intriguingly this article bridges GPCR signaling modulation to specific composition of gut microbiota, and highlights the insufficiently understood role of gut microbes in regulation of pathophysiological processes in humans.

As Guest Editors, we extend our heartfelt gratitude to all the authors for their remarkable commitment, diligence, and outstanding contributions to this special edition of Frontiers in Endocrinology “Advanced Biophysical and Biochemical Technologies to study GPCR Signal Transduction“. We anticipate that this Research Topic will emerge as an important reference for medicinal chemists, chemical biologists, structural biologists, pharmacologists, and fellow researchers involved in or passionate about GPCR drug discovery and development. Authors’ efforts have undoubtedly enriched the scholarly landscape in this field, and we are confident that this compilation will significantly benefit the scientific community.

## Author contributions

MM: Writing – original draft. AB: Writing – review & editing.
